# Curcumin derivative, 2,6-bis(2-fluorobenzylidene)cyclohexanone (MS65) inhibits interleukin-6 production through suppression of NF-κB and MAPK pathways in histamine-induced human keratinocytes cell (HaCaT)

**DOI:** 10.1186/s12906-018-2223-8

**Published:** 2018-07-16

**Authors:** Nurul Atika Razali, Nur Amiza Nazarudin, Kok Song Lai, Faridah Abas, Syahida Ahmad

**Affiliations:** 1 Department of Biochemistry, Faculty of Biotechnology and Biomolecular Sciences, Universiti Putra Malaysia, 43400 Serdang, Selangor Malaysia; 2 Department of Cell and Molecular Biology, Faculty of Biotechnology and Biomolecular Sciences, Universiti Putra Malaysia, 43400 Serdang, Selangor Malaysia; 3 Department of Food Science, Faculty of Food Science & Technology, Universiti Putra Malaysia, 43400 Serdang, Selangor Malaysia

**Keywords:** Histamine, Interleukin-6, Curcumin derivative, Keratinocytes

## Abstract

**Background:**

Histamine is a well-known mediator involved in skin allergic responses through up-regulation of pro-inflammatory cytokines. Antihistamines remain the mainstay of allergy treatment, but they were found limited in efficacy and associated with several common side effects. Therefore, alternative therapeutic preferences are derived from natural products in an effort to provide safe yet reliable anti-inflammatory agents. Curcumin and their derivatives are among compounds of interest in natural product research due to numerous pharmacological benefits including anti-inflammatory activities. Here, we investigate the effects of chemically synthesized curcumin derivative, 2,6-bis(2-fluorobenzylidene)cyclohexanone (MS65), in reducing cytokine production in histamine-induced HaCaT cells.

**Methods:**

Interleukin (IL)-6 cytokine production in histamine-induced HaCaT cells were measured using enzyme-linked immunosorbent assay (ELISA) and cytotoxicity effects were determined using 3-(4,5-dimethylthiazol-2-yl)-2,5-diphenyltetrazolium bromide (MTT) assay. Real-time polymerase chain reaction (RT-qPCR) was carried out to determine the inhibitory effects of MS65 on nuclear factor-kappa B (NF-κB) and mitogen activated protein kinase (MAPK) pathways.

**Results:**

Histamine enhanced IL-6 production in HaCaT cells, with the highest production of IL-6 at 97.41 ± 2.33 pg/mL after 24 h of exposure. MS65 demonstrated a promising anti-inflammatory activity by inhibiting IL-6 production with half maximal inhibitory concentration (IC_50_) value of 4.91 ± 2.50 μM and median lethal concentration (LC_50_) value of 28.82 ± 7.56 μM. In gene expression level, we found that MS65 inhibits NF-κB and MAPK pathways through suppression of IKK/IκB/NFκB and c-Raf/MEK/ERK inflammatory cascades.

**Conclusion:**

Taken together, our results suggest that MS65 could be used as a lead compound on developing new medicinal agent for the treatment of allergic skin diseases.

## Background

Allergic skin diseases generate considerable dermatologist concern due to their increases in prevalence, severity, and complexity [1]. Histamine, a biological amine, has been identified as a major mediator of inflammation and allergic response that regulates the expression of cytokine, chemokines and cell-adhesion molecules [2–4]. The release of histamine in the skin causes a variety of allergic reactions which include redness, itching as well as wheal and flare due to vasodilation and increase in vascular permeability [5]. The exposure of histamine to keratinocytes, the main cells of epidermis (outermost layer of the skin), lead to the formation of an impaired skin barrier, which initiates the expression of proinflammatory molecules that represents the starting point of primary skin inflammation [6, 7].

Histamine exerts its proinflammatory effects mainly through four different receptors (H1, H2, H3 and H4), but mediated mostly by the activation of H1 receptors (H1Rs) in allergic diseases [8]. The binding of histamine to H1R results in phosphorylation of protein kinase C (PKC) and downstream activation of NF-κB and MAPK transcription factors, which are associated with regulation of adhesion molecules, chemotaxis, antigen presentation and proinflammatory cytokine production [9, 10]. IL-6 cytokine is elevated in most inflammatory conditions and have been recognized as targets of therapeutic intervention [11]. The production of IL-6 in human epidermal keratinocytes has been widely studied due to its prominent role in various inflammatory skin diseases. In recent years, there is evidence on IL-6 production in human keratinocytes stimulated by a wide range of inducers such as IL-α, toll-like receptor (TLR) ligands and ultraviolet (UV) radiation [12, 13]. Interestingly, Matsubara et al. [10] has demonstrated the upregulation of IL-6 expression by keratinocytes in response to histamine exposure, suggesting that IL-6 is involved in the pathogenesis of skin allergies.

Considering the roles of H1R in mediating proinflammatory effects of histamine, the therapeutic intervention in allergic disorders has thus commonly focused on developing the antagonists of this receptor [14]. H1 antihistamines, also called H1 antagonists, remain as first-line medications for the treatment of allergic diseases due to their effectiveness in providing symptomatic relief [15]. The action of H1 antihistamines are associated with the suppression of cytokines, chemokines and adhesion molecules transcription [10, 16]. However, increasing evidence have shown that administration of H1 antihistamines is limited in efficacy and associated with a number of side-effects such as nausea, lightheadedness, drowsiness, headaches, agitation and dry mouth [15, 17]. Thus, the discovery of alternative anti-inflammatory agents that are more effective and safe for treatment of allergic skin disorders is of utmost important.

On natural preference of the treatment, a wide spectrum of phytochemicals and their derivatives have been identified for their potential as anti-inflammatory agents. Curcumin and its derivatives have attracted increasing interest due to numerous pharmacological benefits such as anticancer, antioxidant, antimalarial and anti-inflammatory activities [18]. The anti-inflammatory effects of curcumin are mainly mediated through regulation numerous transcription factors, cytokines, protein kinases, adhesion molecules, redox status and enzymes that have been linked to inflammation [19]. The versatility and flexibility for structural modification of natural and synthetic derivatives of curcumin have been explored extensively for designing new medicinal agents with improved potency and lesser toxicity [18]. Therefore, the aim of this work was to investigate the potential inhibitory effects of synthesized curcumin derivative (MS65) on IL-6 production in histamine-induced HaCaT cell line.

## Methods

### Chemicals and reagents

Curcumin derivative, 2,6-bis(2-fluorobenzylidene)cyclohexanone (MS65) was obtained from Laboratory of Natural Products, Institute of Bioscience (UPM) in a form of powder, which was dissolved in 100% dimethyl sulfoxide (DMSO) purchased from Qrec (Asia) Sdn. Bhd. (Selangor, Malaysia). Histamine and Dulbecco’s Modified Eagle’s Medium (DMEM) were purchased from Sigma-Aldrich Co. (St. Louis, MO, USA). Fetal bovine serum (FBS) and penicillin/streptomycin were purchased from PAA Laboratories GmbH (Austria). Levocetirizine dihydrochloride (H1 antihistamine) was purchased from Abcam (Cambridge, MA, USA) and 3-(4,5-dimethylthiazol-2-yl)-2,5-diphenyltetrazolium bromide (MTT) reagent was purchased from Fluka Analytical (St. Louis, MO, USA). Phosphate buffered saline (PBS) tablets were purchased from Amresco Inc. (Solon, OH, USA). All the primers were obtained from First BASE Laboratories Sdn. Bhd. (Selangor, Malaysia).

### Cell culture and induction

HaCaT cells obtained from AddexBio (San Diego, CA, USA) were cultured in DMEM supplemented with 10% FBS and 1% penicillin/streptomycin in a 5% CO_2_ incubator at 37 °C. HaCaT cells (2 × 10^4^ cells/well) were then incubated in 96-well plates with various concentrations of histamine (0.1, 1, 10 and 100 μM) at different incubation hours (12, 24, 36 and 48 h). Cell culture supernatants were collected and kept in − 20 °C for determination of IL-6 production.

### Cell treatments

HaCaT cells (2 × 10^4^ cells/well) were induced with histamine and treated with MS65 (50 μM) and H1 antihistamine (20 μM) in two-fold serial dilutions for 24 h at 37 °C in fully humidified air incubator. Cell culture supernatants were then collected and analysed for IL-6 production.

### Cytotoxicity assay

Cell viability was assessed using MTT assay, in which culture media of histamine-induced HaCaT cells and MS65-treated HaCaT cells were removed and replaced with 5 mg/mL MTT reagent. The cells containing MTT solution were then further incubated at 37 °C for 4 h before removing the solutions. The formazan crystals formed were dissolved in DMSO and absorbance was read at 570 nm using a microplate reader.

### Measurement of IL-6 production

Enzyme-linked immunosorbent assay (ELISA) was performed using kit from eBioscience (San Diego, CA, USA). Briefly, ELISA plate (96-well) was coated with coating buffer containing capture antibody and incubated overnight at 4 °C. After washing with PBS containing 0.05% tween-20, the plate was blocked with assay diluent for 1 h at room temperature (RT). Samples and IL-6 standards were then added to the respective wells in the plate and further incubated for 2 h at RT. The plate was washed and detection antibody was added to each well for 1 h at RT. The washing step was repeated followed by addition of enzyme and incubated for 30 min at RT. The plate was then washed and substrate solution was added to each well for 15 min at RT in the dark. Stop solution was added to each well and absorbance was immediately read at 450 nm.

### RNA extraction and reverse transcription

HaCaT cells were harvested and total RNA was extracted using RNeasy® Plus Mini kit from Qiagen (Hilden, Germany). The concentration and purity of RNA was determined using a NanoDrop 2000c UV-Vis Spectrophotometers from Thermo Fisher Scientific (Waltham, MA, USA). cDNA was prepared from 500 ng of total RNA using 5× iScript Reverse Transcription Supermix for RT-qPCR from Bio-Rad Laboratories (Hercules, CA, USA). The list of primers used in this study is shown in Table 1.

**Table 1:** Primers sequences of target genes

Genes		Primer sequences (5′-3′)	Accession number
H1R	F R	CTG GTT TCT CTC TTT TCT GTG GGT T GAT CTT GGC ATA GAA CCA GAG CAT G	NM_001098213.1
PKC	F R	CAG GCA GAA ATT CGA GAA AGC CAA A TCC CAA CAC CAT GAG GAA ATT GAA G	NM_002737.2
IKK-β	F R	ATC CCC GAT AAG CCT GCC A CTT GGG CTC TTG AAG GAT ACA G	NM_001556.2
IκB-α	F R	ATT GCT GAG GCA CTT CTG GGA GCT G AGA CAC GTG TGG CCA TTG TAG TTG G	NM_020529.2
NF-κβ	F R	GGA CCG CTG CAT CCA CAG TTT CCA G TGT CAC CTG GAA GCA GAG CCG CAC A	NM_001145138.1
c-Raf	F R	CAG TAT CTG GGA CCC AGG AGA AAA A TAG GAT CTT TAC TGC AAC ATC TCC G	NM_002880.3
MEK	F R	CGT ACA TCG TGG GCT TCT ATG GTG C GCA TGA TCT TGT GCT TCT CCC TCA G	NM_002755.3
ERK	F R	CTC TAC CAG ATC CTC AGA GGG TTA A TAC CAA CGT GTG GCC ACA TAT TCT G	NM_002745.4
GAPDH	F R	CAG CCT CAA GAT CAT CAG CA CAT CCA CAG TCT TCT GGG TG	NM_002046.5

*H1R* histamine H1-receptor, *PKC* protein kinase, *IKK-β* inhibitor of nuclear factor kappa-B kinase subunit beta, *IκB-α* Nuclear factor of kappa light polypeptide gene enhancer in B-cells inhibitor, alpha, *NF-κβ* nuclear factor kappa-light-chain-enhancer of activated B cells, *c-Raf* RAF proto-oncogene serine/threonine-protein kinase, *MEK* mitogen-activated protein kinase kinase, *ERK* extracellular signal–regulated kinases, *GAPDH* glyceraldehyde 3-phosphate dehydrogenase

### Real-time polymerase chain reaction (RT-qPCR)

Real-time quantitative PCR was performed on a real time PCR instrument, Bio-Rad® CFX96™. In this assay, iTaq™ Universal SYBR® Green Supermix (2×) kit from Bio-Rad Laboratories (Hercules, CA, USA) was used to determine genes expressions of H1R, PKC, IKK-β, IκB-α, NF-κB, c-Raf, MEK and ERK in histamine-induced HaCaT cells treated with MS65. Reaction setup of mastermix preparation was performed according to manufacturer’s protocol. In brief, assay mastermix was prepared by adding all required components (except cDNA template) according to the suggested volume of total 10 μL per reaction. The 9.5 μL of reaction mixtures was equally distributed into each recipient PCR tubes in triplicate prior to addition of 0.5 μL cDNA template. As for thermal cycling set up, the following PCR settings were used: an initial activation 95 °C for 3 min, followed by denaturation 95 °C for 10 s and annealing/extension at respective temperatures for 30 s (40 cycles). Then, samples were gradually heated from 70 °C to 95 °C with a ramp rate of 0.5 °C/s to obtain melting curves and fusion temperatures of the amplicons.

Kinetic analysis was measured by normalizing amplification threshold cycle (C_T_) values of template samples with C_T_ values of template reference gene (GAPDH). RT-qPCR results were analyzed via relative quantification whereby expressions levels of samples were analyzed in relative amount (fold differences). In this study, induced control was used as calibrator and all samples were analyzed as increased or decreased folds in relative to calibrator by using Livak method [20] with calculation formula of 2^-∆∆CT^ (normalized expression ratio).

The formula was derived from;

$$ {\displaystyle \begin{array}{c}{\Delta \mathrm{C}}_{\mathrm{T}\left(\mathrm{Sample}\right)}={\mathrm{C}}_{\mathrm{T}\left(\mathrm{Target}/\mathrm{Sample}\right)}-{\mathrm{C}}_{\mathrm{T}\left(\mathrm{Reference}/\mathrm{Sample}\right)}\\ {}{\Delta \mathrm{C}}_{\mathrm{T}\left(\mathrm{Calibrator}\right)}={\mathrm{C}}_{\mathrm{T}\left(\mathrm{Target}/\mathrm{Calibrator}\right)}-{\mathrm{C}}_{\mathrm{T}\left(\mathrm{Reference}/\mathrm{Calibrator}\right)}\\ {}{\Delta \Delta \mathrm{C}}_{\mathrm{T}}={\Delta \mathrm{C}}_{\mathrm{T}\left(\mathrm{Sample}\right)-}{\Delta \mathrm{C}}_{\mathrm{T}\left(\mathrm{Calibrator}\right)}\end{array}} $$

### Statistical data analysis

All the results obtained were presented as mean ± standard error of mean (S.E.M) of three independent experiments unless otherwise stated. The differences between groups were determined by using one-way analysis of variance (ANOVA) followed by Dunnett test. The values of *P < 0.05, **P < 0.01 and ***P < 0.001 were considered significantly different from control group. Log IC_50_ calculations were performed using the built-in algorithms for dose-response curves with variable slope using Graphpad Prism software. All graphs in this study were generated by using GraphPad Prism version 7.0 (GraphPad Software, Inc.).

## Results

### Effect of histamine on cells viability and IL-6 production in HaCaT cells

**Fig. 1:**
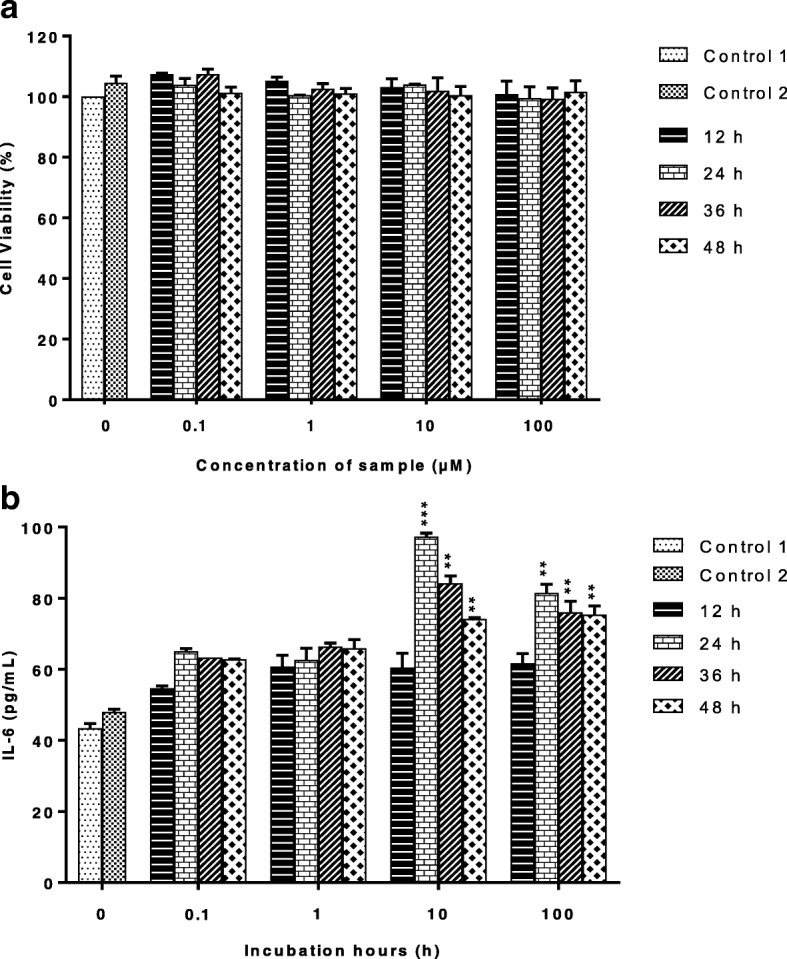
Effects of histamine on (**a**) cells viability and (**b**) IL-6 production in HaCaT cells. Cells were tested in the presence or absence of histamine. Cells were seeded for 24 h before inducing with different concentrations of histamine (0.1-100 μM). Cells were then further incubated for 0-48 h. C1; uninduced cells in DMEM only. C2; uninduced cells with 0.1% DMSO was set as negative control. All values are the mean ± S.E.M. of three independent experiments. Values of **P < 0.01 and ***P < 0.001 were considered significantly different to uninduced control group (C2)

We first evaluate the effect of histamine on HaCaT cells viability using MTT assay. Result shows that histamine demonstrated non-significant cytotoxic effect at all concentrations tested with cells viability more than 90% (Fig. 1a). Next, we examined whether histamine stimulates IL-6 production in HaCaT cells using an ELISA. As shown in Fig. 1b, histamine enhances IL-6 protein expression, with the highest production of 97.41 ± 1.65 pg/mL measured at concentration of 10 μM after 24 h incubation.

### MS65 inhibits IL-6 production in histamine-induced HaCaT cells

**Fig. 2:**
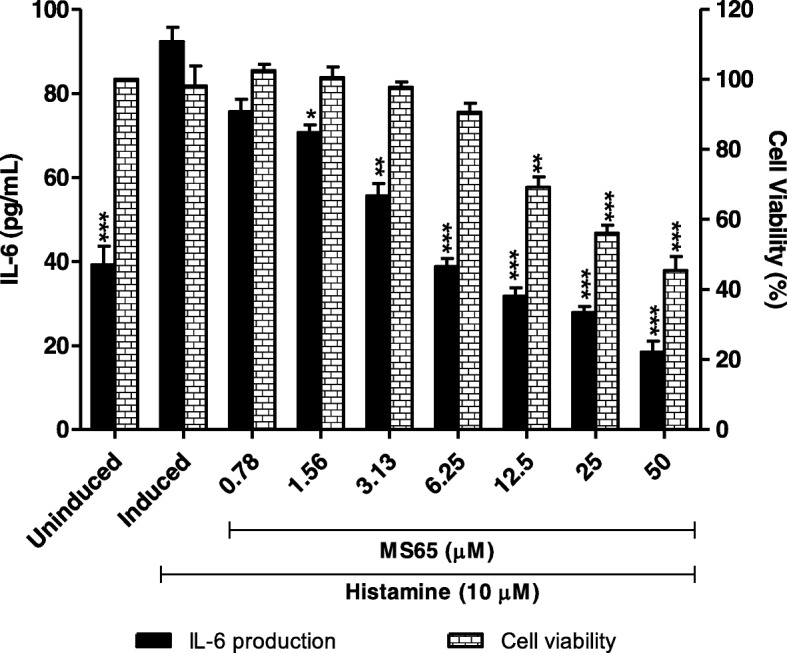
Effects of curcumin derivative, MS65 on IL-6 production and cells viability in histamine-induced HaCaT cells. Cells were seeded for 24 h before inducing with 10 μM of histamine. MS65 was tested in two-fold serial dilution with the highest concentration of 50 μM. Cells were then further incubated for 24 h. All values are the mean ± S.E.M. of three independent experiments. Values of *P < 0.05, **P < 0.01 and ***P < 0.001 were considered significantly different to histamine-induced control group

In order to evaluate the anti-inflammatory activity of MS65, we examined the effect of MS65 on IL-6 production and HaCaT cells viability in two-fold serial dilutions. As shown in Fig. 2, IL-6 production was dose-dependently down-regulated by MS65, in which MS65 showed inhibition at all concentrations tested with IC_50_ value of 4.91 ± 2.50 μM. The cytotoxicity assay shows that MS65 demonstrated non-significant cytotoxicity effect at low concentrations tested (0.78, 1.56 and 6.25 μM); however, HaCaT cell viability started to decrease at concentration of 12.5 μM (69.20 ± 5.06%), followed by 25 μM (56.09 ± 3.95%) and 50 μM (45.42 ± 6.93%), suggesting that MS65 at concentration of 6.25 μM and below were safe to be used on HaCaT cells due to high cell viability (more than 80%).

### H1 antihistamine inhibits IL-6 production with high cytotoxicity effects in histamine-induced HaCaT cells

**Fig. 3:**
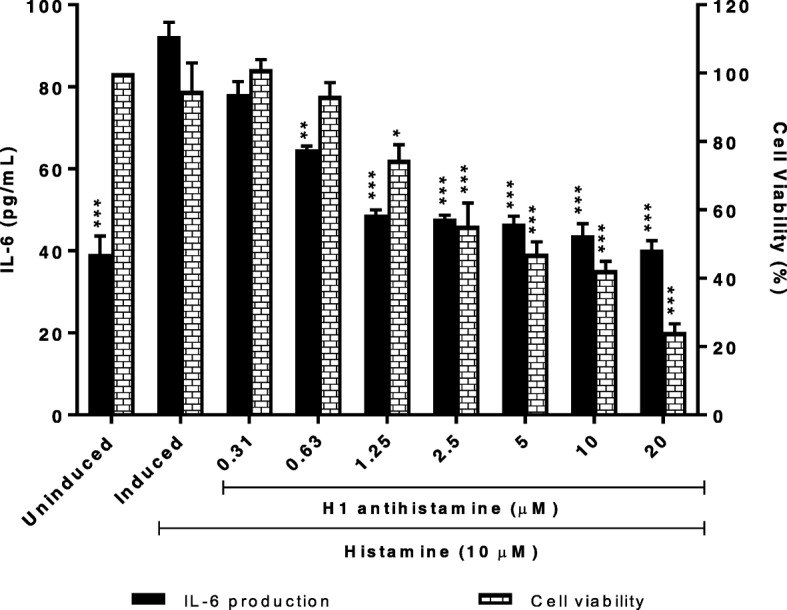
Effects of H1 antihistamine on IL-6 production and cells viability of histamine-induced HaCaT cells. Cells were seeded for 24 h before inducing with 10 μM of histamine. H1 antihistamine was tested in two-fold serial dilution with the highest concentration of 20 μM. Cells were then futher incubated for 24 h. All values are the mean ± S.E.M. of three independent experiments. Values of *P < 0.05, **P < 0.01 and ***P < 0.001 were considered significantly different to histamine-induced control group

In this study, the effect of H1 antihistamine on IL-6 production and HaCaT cells viability were evaluated as a comparison with MS65. As shown in Fig. 3, IL-6 production was dose-dependently down-regulated by H1 antihistamine at all concentrations tested with IC_50_ value of 1.91 ± 1.80 μM. In cytotoxicity assay, result shows that H1 antihistamine demonstrated significant cytotoxicity effect at concentration of 2.5 μM and above. Therefore, it can be suggested that H1 antihistamine demonstrated lower IL-6 inhibition and higher cytotoxicity effects (LC_50_ value of 2.90 ± 1.50 μM) compared to MS65 (LC_50_ value of 28.82 ± 7.56 μM).

### MS65 suppresses gene expression of H1R, PKC, IKK-β, IκB-α, NF-κβ, c-Raf, MEK and ERK

**Fig. 4:**
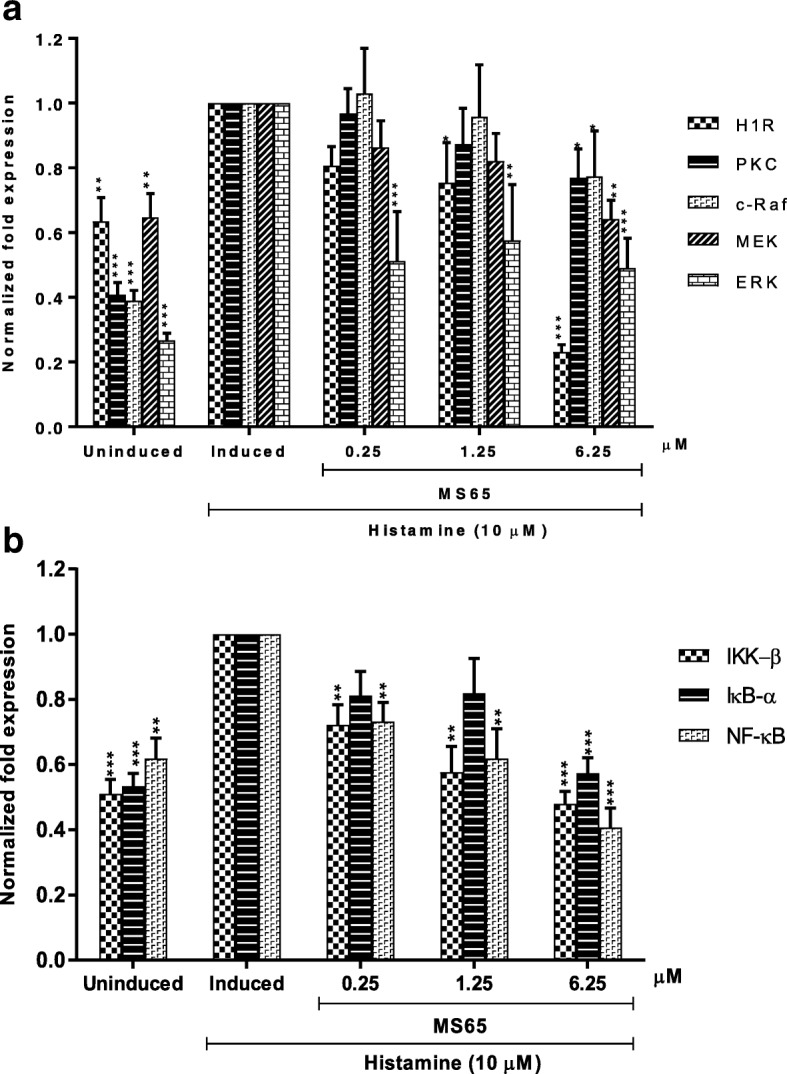
Effects of MS65 on genes expression of (**a**) H1R, PKC, c-Raf, MEK, ERK and (**b**) IKK-β, IκB-α, NF-κβ. Cells were treated with 10 μM of histamine for 24 h in presence or absence of MS65. Total mRNA was extracted, and gene expression was analyzed by RT-qPCR. The fold expressions were normalized by GAPDH expression levels. Data are expressed as the mean ± S.E.M. of three separate experiments. Values of *P < 0.05, **P < 0.01 and ***P < 0.001 were considered significantly different to histamine-induced control group

In order to determine the effects of MS65 on H1R-dependent NF-κβ and MAPK pathway, we evaluated the gene expression of H1R, PKC, IKK-β, IκB-α, NF-κβ, c-Raf, MEK and ERK in MS65-treated histamine-induced HaCaT cells in five-fold serial dilutions (0.25, 1.25 and 6.25 μM) (Fig. 4a and b). In this study, histamine-induced HaCaT cells treated with MS65 showed decrement on all the genes tested with highest decrement at concentration of 6.25 μM. H1R gene demonstrated highest reduction of gene expression at 6.25 μM (0.23 ± 0.02 fold expression), followed by NF-ĸB (0.41 ± 0.06 fold expression), IKK-β (0.48 ± 0.04 fold expression), ERK (0.49 ± 0.09 fold expression), IκB-α (0.57 ± 0.05 fold expression), MEK (0.64 ± 0.06 fold expression), c-Raf (0.77 ± 0.14 fold expression) and PKC (0.77 ± 0.09 fold expression).

## Discussion

Keratinocytes, which comprise 95% of epidermis, are highly active immunological cells with a major control over acute and chronic phases of skin inflammation. During allergies, keratinocytes sense protein allergens and initiate the expression of inflammatory molecules, which represents the starting point of primary skin inflammation. Upon stimulation, keratinocytes are able to secrete a wide spectrum of cytokines, chemokines and accessory molecules, which potently amplifies the innate and adaptive immune responses in the skin [7]. IL-6 cytokine, one of the cytokine secreted by keratinocytes, has been reported to be involved in many inflammatory skin conditions including autoinflammatory and allergic disorders such as psoriasis [21], atopic dermatitis [22] and urticaria [23].

In the present study, we evaluated the effect of histamine on IL-6 production in cultured HaCaT cells. Our study found that histamine enhanced the production of IL-6, adding further evidence about the capacity of this amine to induce cytokine release from keratinocytes. The present result is in agreement with previous studies showing that histamine induces IL-6 production in epidermal keratinocytes [10], lung macrophages [24] and nasal fibroblast [25], indicating that histamine activates a common pathway leading to IL-6 expression in these cells. The enhanced production of IL-6 has been reported to cause wheal formation and rash development, confirming the role of IL-6 in modulating allergic inflammation in skin [13].

In recent years, curcumin and its derivatives have demonstrated promising anti-inflammatory activities by inhibiting several important inflammatory mediators such as tumor necrosis factor (TNF)-α, IL-6, IL-1β, nitric oxide (NO) and nitric oxide synthase (iNOS) [26, 27]. The therapeutic potentials of curcumin are attributed mainly to its chemical structure, in which the presence of various substituents such as methoxy, hydroxyl, alkyl, halogens, amino, nitro, nitril, acetamido, carboxyl, benzene, heterocyclic and condensed rings in their structure may influence their specific biological activities to a certain degree [28, 29]. In pharmacological evaluation, the halogen substitution has been found to enhance anti-inflammatory potency of the compound. Vairappan et al. [30] has demonstrated the anti-inflammatory activity of halogenated secondary metabolites through inhibition of TNF-α, IL-1β, IL-6, and NO production. Several studies also showed that synthesized products with halogens on their aromatic ring favor anti-inflammatory activities [31, 32]. These reports were consistent with our study, whereby halogen-containing curcumin derivative MS65 showed promising anti-inflammatory activity by significantly inhibited IL-6 production in HaCaT cells.

Recent study has shown that structural modification of curcumin enhanced its anti-inflammatory activity by improving the stability in aqueous solutions at physiological pH [33]. The incorporation of halogen atoms causes an increase in thermal and oxidative stability of the compound, resulting in a lack of responsiveness towards oxidation by the liver P450 detoxification. Halogen-containing compounds are also more lipophilic and less water soluble, thus providing better penetration of lipid membranes [34]. Most of halogenated drugs are fluorine drugs, followed by chlorine, while bromine is rare and the only iodine drug is the thyroid hormone thyroxine [35]. In relation to our study, MS65 contains fluorine atoms, which are believed to play a remarkable role in improving its bioavailability. The small and highly electronegativity fluorine atom can modify electron distribution in the molecule, affecting its absorption, distribution and metabolism [36]. Therefore, the fluorinated compounds could have improved metabolic stability, binding affinity and physicochemical properties, leading to better bioavailability [37, 38].

Levocetirizine dihydrochloride is a third generation H1 antihistamines used for the treatment of various allergies including atopic dermatitis and urticarial [39]. In this study, levocetirizine was used as a comparison with MS65. Levocetirizine contains different halogen atoms, which is chlorine, therefore it may have different efficiency than MS65. Different from fluorine, chlorine is a moderate halogen bond acceptor, besides being larger in size than fluorine. However, the C-Cl bond is enough stable, allowing its insertion on diverse heterocyclics of pharmacological value [35]. In this study, we found that histamine-induced IL-6 production was dose-dependently down-regulated by H1 antihistamine and MS65, with the highest inhibition measured at the highest concentrations tested, 20 μM and 50 μM respectively. However, the cytotoxicity assay shows that MS65 is functional at non-cytotoxic levels even though its effective dose range appears to be higher than H1 antihistamine (1.56-50 μM versus 0.63-1.25 μM), suggesting that MS65 is more effective as well as safe and well tolerated to be used than levocetirizine. These findings also raise the intriguing possibility that levocetirizine is unsuitable for a long-term treatment and drug overdose could result in serious health consequences. This assumption is supported by previous study which has reported the first case of levocetirizine-induced hepatotoxicty in a patient with chronic urticarial [40]. Additionally, recent studies have documented few cases of fixed drug eruption (FDE) due to levocetirizine that was given to the patient for allergic rhinitis and scabies [41, 42].

Further investigation is prompted in order to investigate the effect of MS65 on gene expression levels. Reviews of literature have demonstrated the regulation of H1 receptor on cytokine production via downstream activation of NF-κB and MAPK pathways [10, 25]. Matsubara et al. [10] has proposed the underlying mechanism of H1R-dependent NF-κB and MAPK pathways in human epidermal keratinocytes, which involves the phosphorylation of PKC. The coupling of PKC with H1R signalling has been reported in epidermal keratinocytes [10], chinese hamster ovary (CHO) cells [43] and HeLa cells [44]. Briefly, the phosphorylation of PKC by histamine activates IKK to phosphorylate IκB, allowing active NF-κB dimer to translocate into nucleus. The phosphorylated PKC was also involved in the phosphorylation of c-Raf, which further phosphorylates MEK, a MAPKK upstream of ERK/MAPK pathway [10]. In the present study, the effects of histamine and MS65-treated histamine-induced cells were evaluated on gene expression of H1R, PKC, IKK-β, IκB-α, NF-κβ, c-Raf, MEK and ERK in HaCaT cells. As previously reported by Matsubara et al. [10], the stimulation of H1R, PKC, IKK/IκB/NFκB and c-Raf/MEK/ERK inflammatory cascades by histamine confirmed the involvement of NF-κB and MAPK pathways in histamine-induced physiological responses. The treatment of histamine-induced keratinocytes with MS65 demonstrated gene suppression in dose-dependent manner for all the genes tested, with the highest inhibition on H1R gene at the highest concentration of 6.25 μM. H1R expression levels are closely related with the severity of allergic symptoms, therefore, compounds that suppress the up-regulation of H1R gene may found effective in alleviating the allergic symptoms [45].

## Conclusion

Our present study demonstrated that MS65 interacts with H1R gene, thereby blocking the phosphorylation of PKC, allowing the suppression of IKK/IκB/NFκB and c-Raf/MEK/ERK inflammatory cascades as well as preventing their translocation into the nucleus. Therefore, it is attractive to speculate that down-regulation of NF-kB and MAPK pathways may represent a possible mechanism for MS65 to inhibit the IL-6 production in HaCaT cells. Hence, this study may provide a starting point for understanding the potentials of MS65 as an add-on drug in allergic-mediated anti-inflammatory therapies.

## Abbreviations

ANOVA: Analysis of variance
cDNA: Complementary deoxyribonucleic acid
c-Raf: RAF proto-oncogene serine/threonine-protein kinase
DMEM: Dulbecco’s modified eagle medium
DMSO: Dimethyl sulfoxide
ELISA: Enzyme-linked immunosorbent assay
ERK: Extracellular signal–regulated kinase
FBS: Fetal bovine serum
FDE: Fixed drug eruption
GAPDH: Glyceraldehyde 3-phosphate dehydrogenase
H1R: Histamine H1 receptor
HaCaT: Human adult low calcium high temperature
IC_50_: Half-maximal inhibitory concentration
IKK: Inhibitor of nuclear factor kappa-B kinase
IL: Interleukin
iNOS: Nitric oxide synthase
IκB: Inhibitor of nuclear factor kappa-B
LC_50_: Half-maximal lethal concentration
MAPK: Mitogen activated protein kinase
MEK/MAPKK: Mitogen-activated protein kinase kinase
MS65: 2,6-bis(2-fluorobenzylidene)cyclohexanone
MTT: 3-[4,5-dimethylthiazol-2-yl]-2,5-diphenyltetrazolium bromide
NF-κB: Nuclear factor kappa-light-chain-enhancer of activated B cells
NO: Nitric oxide
PBS: Phosphate buffered saline
PKC: Protein kinase C
RNA: Ribonucleic acid
RT: Room temperature
RT-qPCR: Quantitative real-time polymerase chain reaction
S.E.M: Standard error of mean
TNF: Tumor necrosis factor
TLR: Toll-like receptor
UV: Ultraviolet
